# Detection of Synchronous Cancers by Fluorodeoxyglucose Positron Emission Tomography/Computed Tomography during Primary Staging Workup for Esophageal Squamous Cell Carcinoma in Taiwan

**DOI:** 10.1371/journal.pone.0082812

**Published:** 2013-11-29

**Authors:** Shih-Hsin Chen, Sheng-Chieh Chan, Yin-Kai Chao, Tzu-Chen Yen

**Affiliations:** 1 Department of Nuclear Medicine, Chang Gung Memorial Hospital, Taoyuan, Taiwan; 2 Department of Thoracic Surgery, Chang Gung Memorial Hospital, Taoyuan, Taiwan; NIH, United States of America

## Abstract

**Aim:**

The aim of this retrospective study was to investigate the ability of fluorodeoxyglucose positron emission tomography/computed tomography (FDG-PET/CT) in the detection of synchronous cancers during staging workup for esophageal squamous cell carcinoma.

**Materials and Methods:**

We performed a retrospective chart review of 426 Taiwanese patients with esophageal cancer who received FDG-PET/CT during their primary staging workup between December 2006 and December 2011. We defined synchronous cancers as those occurring within 6 months of the FDG-PET/CT scan. All of the synchronous lesions were confirmed by histology or imaging follow-up. The study patients were followed for at least 18 months or were censored on the date of last follow-up.

**Results:**

Fifty patients were excluded from analysis because of the presence of distant metastases. Of the remaining 376 patients, 359 were diagnosed with squamous cell carcinoma (SCC). We identified 17 patients with synchronous cancers, and all of them had a diagnosis of SCC. Synchronous head and neck cancers were the most frequent (n=13, 76.4%), followed by gastrointestinal cancers (colon cancer, n=2; hepatocellular carcinoma, n=1), and renal cell carcinoma (n=1). FDG-PET/CT successfully detected 15 synchronous cancers (12 head and neck cancers, 2 colon cancers, and 1 renal cell carcinoma). In contrast, conventional workup detected only 9 synchronous cancers (7 head and neck cancers, 1 hepatocellular carcinoma and 1 renal cell carcinoma). The sensitivity of FDG-PET/CT and conventional workup in detecting synchronous cancers were 88.2% and 52.9% respectively.

**Conclusion:**

The most frequent synchronous lesions in patients with esophageal SCC were head and neck cancers in Taiwan. Our data indicate that FDG-PET/CT is superior to conventional workup in the detection of synchronous tumors during primary staging for esophageal squamous cell carcinoma.

## Introduction

Esophageal cancer is the seventh most common cancer in Taiwanese males [1], and the sixth most frequently diagnosed cancer worldwide [2]. A significant increase in the incidence of esophageal cancer has been recently reported in Taiwan [1]. In 2009, the age-adjusted incidence rate of this malignancy was 12.97 per 100,000 Taiwanese males [1], which is significantly higher than that observed in the US during 2010 (7.74 per 100,000 men) [3]. In light of these data, significant geographic disparities in esophageal cancer burden are evident. 

Esophageal cancer survival rate varies mainly according to the stage in which the malignancy is diagnosed. Patients with stage I disease have a 5-year survival rate of more than 60%, whereas 5-year survival rates are less than 20% in patients with stage IV disease. Overall, the 5-year overall survival rate for esophageal cancer remains disappointingly low (20.5%) [3]. Although therapy with curative intent is generally possible for patients with early-stage esophageal cancer, the presence of synchronous second primary tumors can influence treatment selection. Previous studies have shown that synchronous tumors are not uncommon in patients with esophageal cancer, with an incidence of second primary malignancies ranging between 4.3 and 11.6% [4-10]. 

Fluorodeoxyglucose positron emission tomography/computed tomography (FDG-PET/CT) has better sensitivity than conventional workup in the detection of distant metastases in esophageal cancer and can thus directly influence management decisions [11]. Therefore, FDG-PET/CT is currently recommended by the National Comprehensive Cancer Network guidelines in for the detection of metastatic disease during the staging procedures [12]. However, the clinical impact of FDG-PET/CT in the identification of second primary cancers in patients with esophageal cancer is still unclear.

There are significant differences in the pathogenesis and geographical distribution of the two main histological types of esophageal cancer (i.e., adenocarcinoma and squamous cell carcinoma [SCC]). In Western countries, FDG-PET/CT has been shown to be useful for the detection of second primary tumors in patients with esophageal adenocarcinoma, the most common site being the colon [8-10]. Differently from Western countries where adenocarcinoma is prevalent, SCC is the most common form of esophageal cancer in Asia [13] and the most common second primary tumor site in SCC is in the head and neck region or the stomach [4-7]. Currently, there is a paucity of data on the clinical usefulness of FDG-PET/CT in the detection of synchronous malignancies in Asian patients with esophageal cancer. Therefore, the aim of this retrospective study was to investigate the ability of FDG-PET/CT in the detection of unexpected synchronous neoplasms during the staging workup of Taiwanese patients with primary esophageal SCC.

## Materials and Methods

### Ethics Statements

The Institutional Review Board of the Chang Gung Memorial Hospital approved the study protocol (101-1991B). The requirement of informed consent from the participants was waived because of the retrospective observational nature of this study. The data set were stored securely, accessibly only to the investigator, and analyzed anonymously. 

### Subjects

We performed a retrospective chart review of 426 Taiwanese patients with histology-proven esophageal cancer who received FDG-PET/CT during their primary staging workup between December 2006 and December 2011. Fifty patients were excluded from analysis because of the presence of distant metastases.

### Conventional workup

The conventional workup (CWU) included computed tomography (CT), barium esophagram, and panendoscopy. All of the abnormalities identified during the initial staging and within 6 months thereafter were carefully recorded. A retrospective review of CT images was performed to identify the number of affected lymph nodes and accommodate staging according to the seventh edition of the AJCC Cancer Staging Manual.

### FDG-PET/CT

FDG-PET/CT scans were performed using a GE Discovery ST 16 PET/CT scanner. Patients fasted for at least 4 h before the tracer injections, and scans were obtained 50 min after the intravenous administration of 370 MBq FDG. Non-contrast CT data were acquired for anatomical correlation and attenuation correction. The CT data were acquired at the following settings: 120 kV, automatic mA (ranging from 10 to 300 mA), pitch 1.75:1, collimation 16 × 3.75 mm, and rotation cycle 0.5 s. Whole-body PET scans were performed from the skull base to the mid-thigh, with 3 min of acquisition time per bed position. We used a total of 7-9 cradle positions to enable height adjustment. 

### Image interpretation

FDG-PET/CT images were analyzed by board-certified nuclear medicine physicians. The presence of synchronous cancers in the FDG-PET/CT reports was carefully recorded. Synchronous malignancies were defined by abnormal FDG uptake on FDG-PET unlikely due to distant metastases from the primary esophageal lesion. When possible, all of the synchronous lesions identified by FDG-PET/CT or CWU were confirmed by histology. We used the results of imaging follow-up when histopathological confirmation was not feasible. 

### Treatment

All of the treatment decisions were taken after discussion in the tumor board. A total of 301 patients received concurrent chemoradiotherapy (CCRT). Of them, 186 received definitive CCRT, whereas 115 were treated with CCRT followed by surgery. Sixty-two patients received surgery, and 17 of them were subsequently treated with adjuvant therapies. Five patients received chemotherapy only and 8 refused any treatment.

### Statistical analysis

Descriptive statistics were summarized using percentiles, means, medians, and standard deviations. Categorical variables were analyzed using the chi-square test. Overall survival was calculated from the date of diagnosis until the date of death or censored at the last follow-up. Survival curves were evaluated using the Kaplan- Meier method and compared with the log-rank test. All statistical analyses were performed using SPSS software version 16.0 (SPSS Inc., Chicago, IL, USA). Two-tailed P values <0.05 were considered statistically significant.

## Results

### Patient population

**Figure 1:**
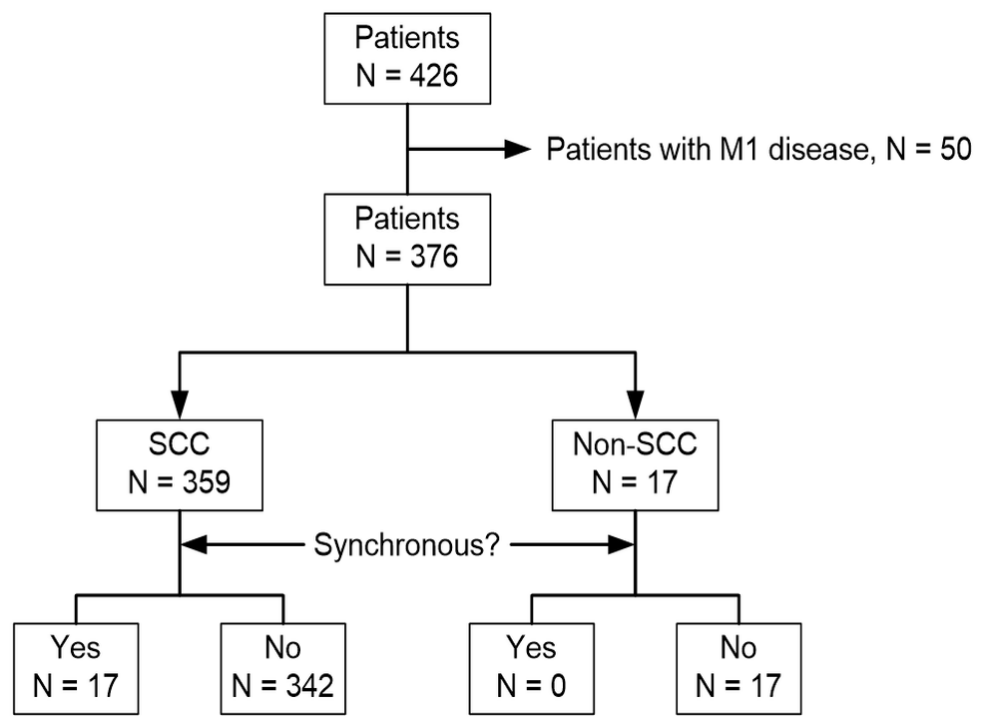
Flow of the patients through the study.

Of the 376 patients without distant metastases, 359 had a diagnosis of SCC (95.5%), and all of the patients with synchronous cancers were diagnosed with this histology type (Figure 1). The median age of these 359 patients was 56 years and female subjects consisted only 5.3% of them (Table 1).

**Table 1:** General characteristics of patients with esophageal squamous cell carcinoma patients according to the presence of synchronous cancers.

	No synchronous cancers (n=342), n (%)	With synchronous cancers, (n=17), n (%)	p
Sex			0.388
Male	323 (94.4)	17 (100.0)	
Female	19 (5.6)	0	
Age, years			0.517
30-39	20 (5.8)	0	
40-49	81 (23.7)	6 (35.3)	
50-59	120 (35.1)	6 (35.3)	
60-69	71 (20.8)	5 (29.4)	
70-79	39 (11.4)	0	
≥ 80	11 (3.2)	0	
Grading^1^			0.584
WD	5 (1.5)	0	
MD	197 (57.6)	8 (47.1)	
PD	74 (21.6)	6 (35.3)	
Unknown	66 (19.3)	3 (17.6)	
Tumor location			0.553
Cervical	19 (5.5)	2 (11.8)	
Upper	95 (27.8)	5 (29.4)	
Middle	148 (43.3)	8 (47.0)	
Lower	80 (23.4)	2 (11.8)	
Stage			0.033
1-2	75 (21.9)	8 (47.1)	
3	267 (78.1)	9 (52.9)	
T-stage			0.034
1-2	76 (22.2)	8 (47.1)	
3-4	266 (77.8)	9 (52.9)	
N-stage			0.006
N0	46 (13.5)	7 (41.2)	
N+	296 (86.5)	10 (58.8)	

^1^ WD = well-differentiated, MD = moderately-differentiated, PD = poorly-differentiated.

Synchronous cancers were diagnosed in 17 patients (17/359, 4.7%). The diagnosis was confirmed by histopathology (n = 15), typical imaging feature of hepatocellular carcinoma (n = 1), or an increase in the colon lesion size on imaging follow-up (n = 1). Synchronous head and neck cancers were the most frequent (n = 13, 76.4%), followed by gastrointestinal cancers (colon cancer, n = 2; hepatocellular carcinoma, n = 1), and renal cell carcinoma (n = 1; table 2). Three patients had premalignant lesions, including colon tubular adenoma (n = 1), hypopharyngeal carcinoma in situ (n = 1), and gastric carcinoma in situ (n = 1).

**Table 2:** List of patients with esophageal cancer diagnosed with synchronous malignancies.

Age, years	Sex	Location	Grade**^1^**	Stage	Second primary**^2^**	Second tumor stage	Detected by CWU**^3^**	Detected by FDG-PET	Alive	Survival, days
60	M	U	PD	2A	Colon	1	N	Y	N	662
56	M	C	MD	3C	Colon	3C	N	Y	N	515
65	M	U	MD	2B	HPX	1	N	Y	N	455
45	M	M	PD	2B	HPX	1	N	Y	Y	755
67	M	L	MD	3B	HPX	1	N	Y	Y	842
51	M	M	MD	3B	HPX	2	N	Y	Y	935
50	M	M	PD	1B	Tongue base	1	N	Y	Y	1574
51	M	L	MD	2A	Tonsil	3	N	Y	Y	899
49	M	C	MD	2B	HPX	1	Y	N	Y	720
62	M	U	Unspecified	3A	HCC	1	Y	N	N	367
58	M	M	MD	3A	HPX	2	Y	Y	N	650
49	M	U	Unspecified	2A	HPX	2	Y	Y	N	667
70	M	M	Unspecified	3B	HPX	3	Y	Y	N	167
42	M	M	PD	3A	Tonsil	3	Y	Y	N	552
48	M	U	MD	3C	Tongue	3	Y	Y	N	535
54	M	M	PD	3C	OPX	4A	Y	Y	N	88
62	M	M	PD	1B	RCC	1	Y	Y	N	95

^1^ MD = moderately differentiated, PD = poorly differentiated.

^2^ HCC = hepatocellular carcinoma, HPX = hypopharynx, OPX = oropharynx, RCC = renal cell carcinoma.

^3^ CWU = conventional workup.

### PET/CT and CWU for the detection of synchronous cancers in patients with esophageal cancer

FDG PET/CT successfully detected synchronous tumors in 15 patients (15/359, 4.2%). The detected lesions included head and neck cancers (n = 12), renal cell carcinoma (n = 1), and colon cancers (n = 2). Of all the synchronous malignancies, six head and neck cancers and two colon cancers were detected by FDG-PET/CT only (47%, 8/17). In contrast, CWU revealed the presence of synchronous tumors in nine patients (9/359, 2.5%), including head and neck cancers (n = 7), hepatocellular carcinoma (n = 1), and renal cell carcinoma (n = 1). Two lesions (11.8%, 2/17) were detected by CWU only, including one case of hepatocellular carcinoma (subsequently confirmed by angiography) and one case of hypopharyngeal cancer (subsequently confirmed by histology). Overall, seven synchronous cancers (6 head and neck cancers and 1 renal cell carcinoma) were detected by both FDG PET/CT and CWU. 

### False-positive findings in the detection of synchronous cancers

Six patients had false-positive PET/CT findings for the presence of synchronous cancers. Of them, two were premalignant lesions (one hypopharyngeal carcinoma in situ and one colon tubular adenoma). Two other patients were diagnosed with squamous hyperplasia of the tongue and the hypopharynx, respectively. Another patient had a left gastric lymph node metastasis which was misinterpreted as a cancer located to the body of the stomach on PET/CT scans. All of these lesions were confirmed by histology. Finally, one patient presenting with asymmetric FDG uptake at the larynx had a diagnosis of vocal palsy at the contralateral side. This condition was diagnosed by fiber endoscopic examination and the results of imaging follow-up were negative. Conventional workup yielded false-positive results in one patient who had a gastric carcinoma in situ (subsequently confirmed by histology).

### Metachronous cancers

At the end of the follow-up, 112 of the 359 study patients were alive. Of them, seven patients developed metachronous cancers (including 6 head and neck cancers and 1 prostate cancer) and were alive at the end of the study. In contrast, other 4 patients who developed metachronous malignancies (3 head and neck cancers and 1 hepatocellular carcinoma) died during the follow-up period. The patient diagnosed with hepatocellular carcinoma also developed a third primary oropharyngeal cancer 243 days after the diagnosis of the liver malignancy.

## Discussion

**Figure 2:**
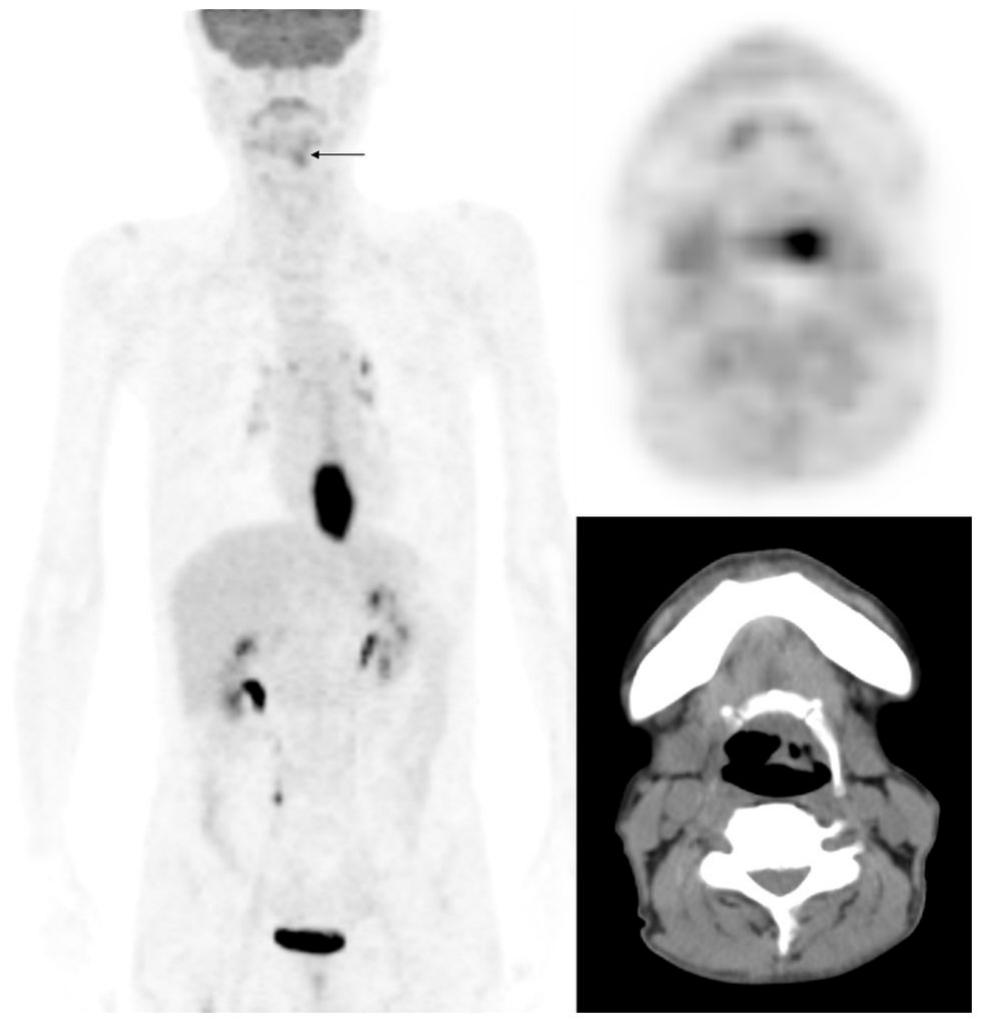
A case of synchronous cancer identified on FDG-PET/CT. In addition to the cancer located in the lower third of thoracic esophagus, FDG-PET/CT identified an additional left hypopharyngeal malignancy missed by conventional CT images (arrow).

In our study conducted among 359 patients with potentially curable esophageal SCC, we identified 17 cases (4.7%) of synchronous cancers. Of them, seven (41%) were correctly identified by both FDG-PET/CT and CWU. Notably, eight (47%) cases were identified by FDG-PET/CT only, whereas two (12%) were diagnosed by CWU only. The sensitivity of FDG-PET/CT in the detection of synchronous cancers was 88.2% (15/17), whereas CWU had a diagnostic sensitivity of 52.9% (9/17). The added diagnostic value of FDG-PET/CT in the detection of synchronous cancers was chiefly confined to the head and neck region (Figure 2). The detection rates of synchronous cancers in the head and neck region were 92.3% and 53.8% for FDG-PET/CT and CWU, respectively. Because the majority of synchronous malignancies in patients with esophageal SCC are head and neck cancers, the observation that FDG-PET/CT is superior to CWU in the detection of these lesions suggests its clinical utility for the clinical management of this patient group.

Notably, FDG-PET/CT was able to detect synchronous colon cancers that are likely to be missed by the CWU. Because colon lesions are not usually investigated during CWU, our findings highlight the utility of FDG-PET/CT as a whole-body screening tool for synchronous malignancies in patients with esophageal SCC [14]. In fact, colon cancer has an increasing incidence trend in Taiwan and has been the most commonly diagnosed cancer in 2009 [1]. In this scenario, the ability of FDG-PET/CT in the detection of a highly prevalent malignancy like colon cancer may have a significant impact on prognostic stratification and clinical management.

**Figure 3:**
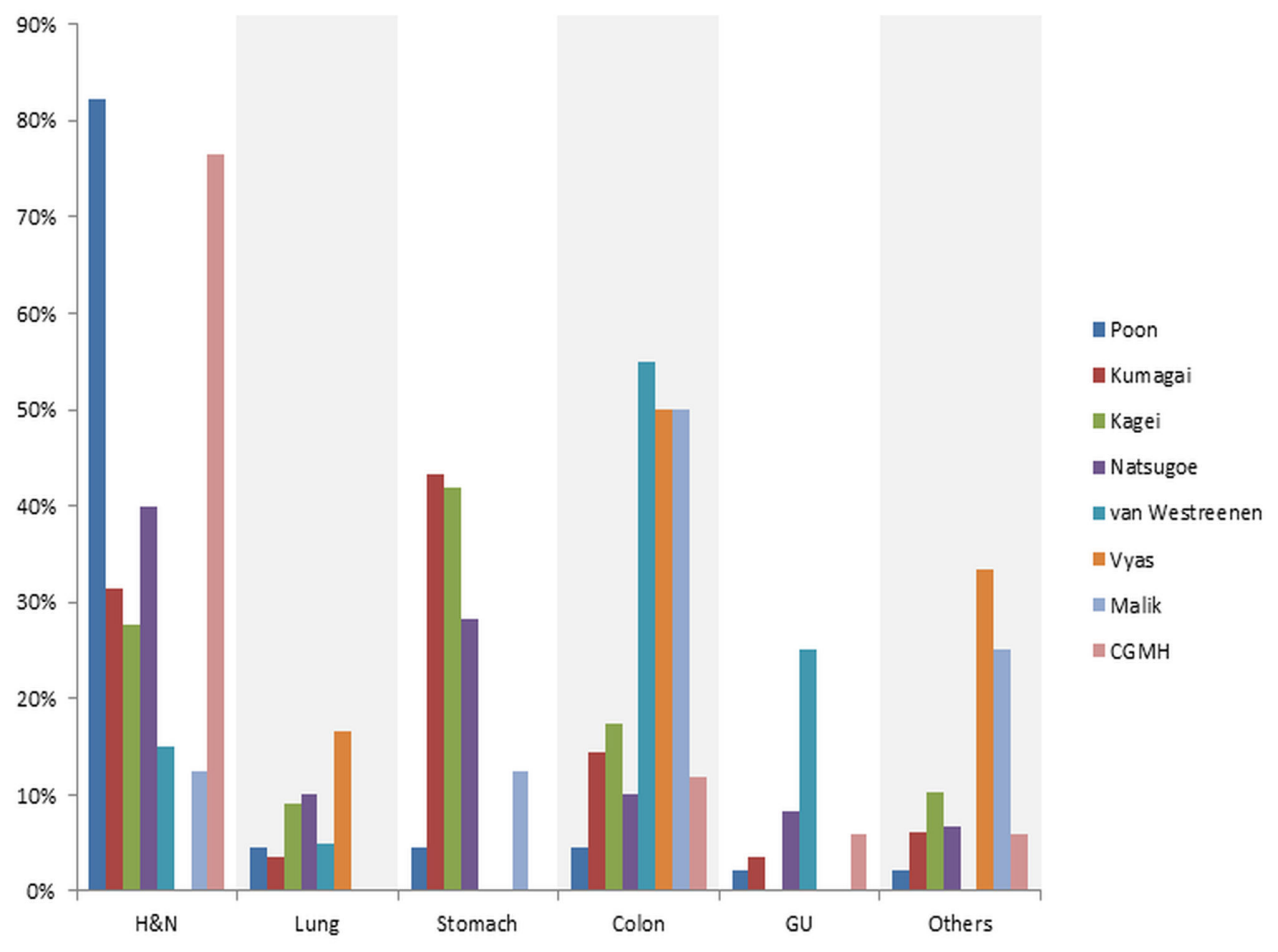
Distribution of synchronous tumors in patients with esophageal cancer according to the published studies. Head and neck cancers either represented the majority of synchronous cancers or was second to stomach cancer in studies conducted in Asian countries, including Hong Kong (Poon), Japan (Kumagai, Kagei, Natusgoe) and Taiwan (CGMH). In contrast, colon neoplasms were the most common form of synchronous tumors in studies conducted Western countries (van Westreenen, Vyas, Malik).

The results from the present study indicate that the most frequent synchronous malignant lesions in patients with esophageal SCC were head and neck cancers. These results are in agreement with those previously reported in studies conducted in Japan and Hong Kong [4-7], where SCC is the main histological subtype of esophageal cancer. In the report of Kumagai et al. and Kagei et al., head and neck cancers were also one of the major types of second primary cancers. Where as in the reports of Poon et al. and Natsugoe et al., head and neck cancers were the leading synchronous cancers (Figure 3). Taken together, studies conducted in Asian countries indicate a close relationship between esophageal and head and neck squamous cell carcinomas, suggesting that these malignancies may be manifestations of field cancerization in the upper aerodigestive tract. Cigarette smoking and alcohol consumption are well-known risk factors for esophageal cancer [15]. An epidemiological study conducted in Taiwan has shown that the odds ratio for esophageal cancer is greater for alcohol drinking than smoking (17.6 vs 5.4) [16]. Moreover, alcohol drinking is a recognized risk factor for head and neck malignancies [17], esophageal cancer [18], stomach cancer [19], and colon neoplasms [20]. The increased cancer risk in people who consume alcohol has been attributed to the first metabolite of alcohol oxidation, acetaldehyde. Acetaldehyde is highly mutagenic and carcinogenic, especially in subjects carrying specific aldehyde dehydrogenase risk polymorphisms [21]. This observation can explain the association of esophageal cancer with both head and neck malignancies and gastrointestinal cancers observed in our study. Moreover, these findings are in accordance with the results from previous investigations conducted in Japan and Hong Kong.

Previous studies on the detection of second primary neoplasms on FDG-PET scans used for staging purposes were conducted in the Netherlands [8], UK [9], and Ireland [10]. The majority of patients with esophageal cancer enrolled in these studies were diagnosed with adenocarcinomas, and the most common site of synchronous tumors was the colon (Figure 3). Differences in the distribution of second primary neoplasms between esophageal SCC and adenocarcinoma indicate the presence of distinct biological features and clinical behavior. To our knowledge, this is the first study assessing the clinical utility of FDG-PET/CT during primary staging of patients with esophageal SCC.

In our study, synchronous cancers occurred more frequently in patients with early-stage esophageal cancer. The median overall survival of patients with and without synchronous cancers was 650 ± 87 days and 390 ± 40 days, respectively. Although the difference between the two groups did not reach statistical significance (log-rank test, p=0.398), the trend toward a longer overall survival in subjects with synchronous cancers can be due at least in part to the association with early-stage disease and prompt initiation of treatment following early diagnosis. The FDG-PET/CT identification of second primary synchronous cancers in patients with early-stage esophageal cancer may thus translate into a potential survival benefit.

Besides being the most common synchronous cancers identified during primary staging, head and neck cancers also represented the majority of metachronous cancers in esophageal SCC patients. Of the 11 metachronous cancers that occurred during the follow-up period, 9 were head and neck cancers; these results confirm the existence of a strong association between esophageal SCC and head and neck cancers. Because metachronous cancers may develop even five years after the diagnosis of the primary cancer, long-term follow-up studies are needed to confirm and expand our results. 

Despite the promising results in the identification of potentially curable synchronous cancers, FDG-PET yielded a certain degree of false-positive and false-negative findings. Because FDG is a non-specific tracer, its uptake does not always indicate the presence of malignant lesions. For example, an increased physiologic uptake of FDG may be commonly observed in the gastroesophageal junction [22]. Moreover, false-positive results due to the presence of lymphoid hyperplasia have been also reported in the head and neck region [23]. The misregistration of PET and CT images may also cause difficulties in image interpretation [24]. Under these circumstances, pathological results are needed to avoid inappropriate clinical management. Furthermore, FDG-PET did not yield reliable results in the detection of superficial esophageal lesions. Previous reports found only half of the clinical T1 lesions being correctly detected on FDG-PET scans [25,26]. These caveats notwithstanding, FDG-PET/CT was clinically useful in the identification of early synchronous tumors in potentially curable esophageal SCC patients. Our study provides clues about the usefulness of FDG PET/CT in the staging of patients with esophageal cancer, not only for identifying distant metastases but also for the purpose of detecting second primary cancers.

Our report has several limitations. First, our study is retrospective in nature and a selection bias cannot be excluded because not all patients with esophageal cancer underwent FDG-PET/CT during the staging workup. Second, some synchronous lesions may have disappeared after chemoradiotherapy; consequently, we may have underestimated the exact number of lesions. It should be noted, however, that our data are in line with those previously obtained in previously published reports from Asian countries. We therefore believe that our data are valid and can be clinically helpful to improve the clinical management of Asian patients with esophageal cancer. 

## Conclusions

The most common form of esophageal cancer in Taiwan is SCC, and the most frequent synchronous lesions in this patient group are head and neck malignancies. Our data indicate that FDG-PET/CT is superior to conventional workup in the detection of synchronous tumors (especially head and neck cancers and colon neoplasms) during primary staging for esophageal squamous cell carcinoma. We conclude that FDG-PET/CT may allow early therapeutic interventions and confer survival benefits in this patient group.
